# Combination Use of BMP2 and VEGF165 Promotes Osseointegration and Stability of Titanium Implants in Irradiated Bone

**DOI:** 10.1155/2018/8139424

**Published:** 2018-11-29

**Authors:** Bo Huang, Qianqian Yao, Yan Huang, Liang Zhang, Yang Yao, Ping Gong, Hua Tang

**Affiliations:** ^1^State Key Laboratory of Oral Diseases, West China Hospital of Stomatology, Sichuan University, Chengdu 610041, China; ^2^Dental Implant Center, West China Hospital of Stomatology, Sichuan University, Chengdu 610041, China; ^3^Oral Medical Center, The Second Xiangya Hospital, Central South University, Changsha, China; ^4^OMFS-IMPATH Research Group, Oral Imaging Center, Department of Imaging and Pathology, Biomedical Sciences Group, University of Leuven, Leuven, Belgium

## Abstract

**Background:**

Clinical data demonstrated that failure rate of titanium implant in irradiated bone was 2-3 times higher than that in nonirradiated bone and it is difficult to get the ideal results in irradiated bone.

**Purpose:**

The aim of the study was to investigate the effects of HBO, BMP2, VEGF165, and combined use of BMP2/VEGF165 on osseointegration and stability of titanium implant in irradiated bone.

**Materials and Methods:**

Sixty rabbits were randomly assigned to 5 groups (control group, HBO group, VEGF165 group, BMP2 group, and BMP2/VEGF165 group) after receiving 15 Gy radiation. Implant surgery was performed on tibias eight weeks later. They were sacrificed at two or eight weeks after operation. Implant stability, calcium, and ALP activity in serum, the ratio of bone volume to total volume, the rate of bone growth, and gene expression were assessed.

**Result:**

There was no mortality and no implants failed during the experiment. Implant stability was significantly compromised in the control group compared to the other four experimental groups, and the BMP2/VEGF165 group had the highest implant stability. HBO, BMP2, and VEGF165 significantly increased BV/TV and the rate of bone growth, while the BMP2/VEGF165 showed the best effect among groups. The expression of RUNX2 in HBO, BMP2, and VEGF165/BMP2 group was higher than that in the VEGF165 and control groups at two weeks. The expression of OCN in HBO, BMP2, VEGF165, and VEGF165/BMP2 groups was higher than that in the control group, and the gene expression of CD31 was higher in HBO, VEGF165, and BMP2/VEGF165 groups than that in control and BMP2 groups.

**Conclusion:**

HBO, BMP2, and VEGF165 could increase bone formation around the implant and improved the implant stability in irradiated bone. The combination use of BMP2 and VEGF165 may be promising in the treatment of implant patients with radiotherapy.

## 1. Introduction

Currently, dental implants are considered as an appropriate way to restore the missing teeth [1, 2]. The success of titanium implants is based on the surface morphology of titanium implants, the effective osseointegration, healthy of the peri-implant tissue, and the reestablishment of function [3, 4]. Head and neck cancers are more likely to occur among elderly people worldwide [5]. Radiotherapy is an effective method for the treatment of head and neck cancer, while radiotherapy may cause radioactive bone injury (RBI), osteoradionecrosis, blood vessel fibrosis, the reduction of saliva production and cellular production, mucositis, and other adverse reactions [5–7]. Clinical data demonstrated that failure rate of titanium implant in irradiated bone was 2-3 times higher than that in nonirradiated bone [8, 9]. It was also reported that higher bone resorption could be seen around implants in irradiated areas than nonirradiated areas [10]. Animal experiments showed that the stability of titanium implant and osseointegration were compromised by radiation in a dose-dependent manner [8]. When dogs received 60 Gy radiation, the failure rate of the implant was one hundred percent [8].

The damage of radiation would persist for a long time, and osteoradionecrosis may still occur in ten or twenty years after radiation. To alleviate the damage and increase the success rate of titanium implants, a series of methods, such as hyperbaric oxygen (HBO) and the use of growth factors, were adopted in previous studies. Hyperbaric oxygen therapy could help oxygen diffuse in local tissue, improve bone formation and bone maturation, promote the healing of soft tissue, and reduce failure of titanium implants in irradiated bone. But there are many contraindications in HBO therapy [7, 10, 11], for the risk of pneumothorax tension, claustrophobia, ocular aneurysm, convulsion associated with toxicity of oxygen, and rupture of drum membrane. The time, the cost, and the real necessity of HBO therapy should be clinically considered as well [11, 12]. Other studies reported that HBO provided no additional benefits for improving the success rate of titanium implants in irradiated tissue [7, 13].

As for cellular factors, VEGF165 and BMP2 are the two most commonly used and most effective factors in promoting osseointegration. VEGF165, a heparin-binding growth factor of VEGF family and the most active isoform of VEGF, acts as a mitogen of endothelial cells, a chemotactic mediator, and a vascular permeability inducer [14]. It plays a critical role in recruiting EPCs from the bone marrow, upregulating other angiogenic factors, promoting angiogenesis, and improving the formation of endothelium [15]. Evidence has shown that radiation treatment leads to vascular damage and reduces the expression of VEGF165. Then the process of new blood vessel formation and the ability of vessels to deliver oxygen and nutrients to normal tissues would be seriously interfered, which inhibits the capacity of normal tissue healing and regenerating [15, 16]. BMP, a subfamily of the TGF-beta superfamily and one of the most promising growth factors to enhance bone regeneration, is a group of secreted, hydrophobic, acid glycoproteins that can induce the differentiation of osteoblasts and was originally named for its role in the induction of bone formation [17, 18]. In the BMP family members, BMP2 is the strongest factor for osteogenic induction. During bone regeneration, BMP2 works with other growth factors. Better bone regeneration and vascularization have been reported for the combined use of BMP2 and VEGF165 in comparison to the single use of either BMP2 or VEGF165 in previous literature [19–21]. Dual delivery of the two growth factors for repair of critical size mandibular defects can significantly improve the quantity and quality of early and late bone formation compared to the delivery of rhBMP2 or VEGF alone, indicating that BMP2 and VEGF165 work together to regenerate bone tissue and promote vessel formation [20, 21]. Recent studies assessed the osseointegration capability of titanium implants coated with rhBMP-2 and rhVEGF. The results showed that the combination of rhBMP-2 and VEGF applied locally could enhance the vertical bone generation and improve the quality and quantity of bone around implants in vivo compared to using implants alone, or implants covered with either VEGF165 or rhBMP2 [19].

The combination use of BMP2 and VEGF165 can promote angiogenesis and improve osseointegration around implants. However, whether BMP2 and VEGF165 could increase bone osseointegration around implants or increase success rates of titanium implants in irradiated bone remains unknown. The aim of this research is to study the effect of combined use of BMP2 and VEGF165 on osseointegration around titanium implants and implant stability in irradiated bone.

## 2. Materials and Methods

### 2.1. Animal Care

The animal experiment protocol was approved by the Animal Research Ethics Committee of West China Hospital of Stomatology, Sichuan University (NO. WCCSIRB-D-2014-065). A total of sixty adult male New Zealand White rabbits, weighing 3-4 kg, were divided into five groups. The rabbits were kept in the State National Key Laboratory of Biotherapy.

### 2.2. Radiation

**Figure 1:**
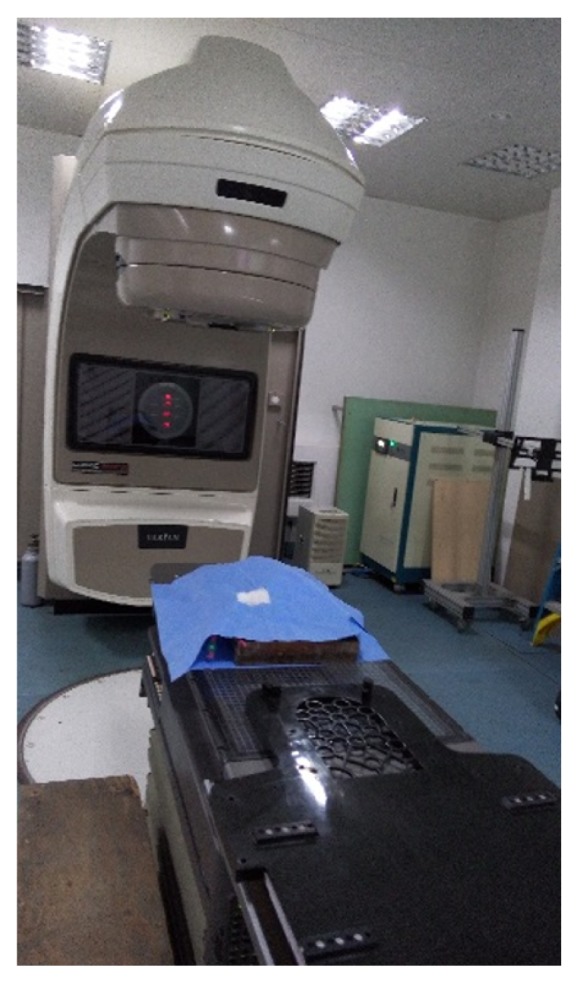
Radiotherapy setup.

Radiotherapy was performed in the Seventh People's Hospital of Chengdu, China. The radiation area was the metaphysis region of the tibia and femur as shown in Figure 1. A single dose of 15 Gy was delivered at a rate of 0.83 Gy/min using a linear accelerator with a source-skin distance of 60 cm and the field of size was 10 × 10 cm^2^.

### 2.3. Implant Surgery

Two months after the radiation, the rabbits received implant surgery. Under general anesthesia, the implants (3.3 × 10 mm, BLB, China) were placed in the tibia 10 mm below the knee joint. The implant sites were prepared according to a standardized procedure. Two implants (one tibia, one implant) were used in one animal. Postoperative antibiotics were administered in the first five days. Lentiviral vector was injected into the prepared holes before implant insertion. The animals were divided into five groups: Group I: empty lentiviral vector; Group II: irradiated rabbits with HBO treatment; Group III: VEGF165 overexpression lentiviral vector; Group IV: BMP2 overexpression lentiviral vector; Group V: BMP2/VEGF165 overexpression lentiviral vector. The concentration of the lentiviral vector is 10^9^/ml. The way to get lentiviral vector has been described in previous studies in our group [22].

### 2.4. HBO Treatment

HBO treatment was carried out in West China School of Public Health No. 4, Sichuan University. After implant surgery, HBO group rabbits received HBO therapy treatments of 100% oxygen at 2.5 atmospheres for 80 min (4 periods of 20 min), five times a week. The activities of rabbits were observed during HBO treatment. The eardrum and the activities of the rabbit were carefully observed after HBO [23].

### 2.5. Detection of Calcium and ALP Activity in Serum

Serum calcium and ALP activities were measured at 1, 3, 7, 14, 28, and 56 days after implantation in West China Hospital of Stomatology, Sichuan University. Blood samples were taken from the ear vein and detected within 20 minutes after blood collection.

### 2.6. Implant Stability Measurement

The torque and primary stability were measured with the torque wrench and the RFA device (Osstell^TM^; Integration Diagnostics, Savedalen, Sweden) immediately after the implant insertion, respectively. Secondary stability and uniaxial pullout test of implants were measured after the animals were killed. The uniaxial pullout test was performed with an electronic universal material testing machine (Instron, USA)

### 2.7. Micro-CT Analyses

In this study, the samples were scanned by using a high-resolution scanner (Micro-CT *μ*CT80, SCANCL Medical, Bassersdorf, Zurich, Switzerland) in the State Key Laboratory of Oral Diseases of Sichuan University at a steep angle of 0.18 over 360°. Measurements were taken at an operating voltage of 101 kVp and 96 mA current and 6 mm isotropic voxel resolution, with an exposure time of 400 ms, and five frames were averaged per view. The region of interest (ROI) was specified as an area with a diameter of 180 um surrounding the implants. Trabecular thickness (Tb.Th), trabecular spacing (Tb.Sp), and the bone volume to total volume (BV/TV) of implant osseointegration were analyzed.

### 2.8. Fluorochrome Labeling Analyses

Alizarin red (30 mg/kg) and calcein green (50 mg/kg) were injected sequentially at week 1 and week 2 after the implant surgery. A total dose volume of 3 ml of each labeling solution was used in each animal. The samples were dehydrated in gradient ethanol and embedded in methyl methacrylate (MMA, Technovit 7500, Kulzer, Hamburg, Germany). The sections were made by using a microtome saw (EXAKT E300CP, Germany). Different kinds of sandpapers were used to get the final thickness of 100 um slides. Fluorescent microscopy (Leica, TCS SP2, Germany) was used to analyze the slides. The average distance between the fluorochromes labeling each day was defined as the bone growth rate.

### 2.9. RT-PCR Analyses

At two and eight weeks, rabbits were sacrificed and bone tissues bordering the implant (1 mm around the implant) were carefully dissected with an annular bone drill. Then the implant was carefully removed. Total RNA was isolated using Trizol reagent (Invitrogen, USA). The detailed procedure for real-time RT-PCR had been described in previous studies of our group [22]. Primer sequences for BMP-2, VEGFA, RUNX2, OCN, CD31, VEGFR, CTSK, IL-6, and P53 were listed in Table 1. GAPDH served as internal control.

### 2.10. Statistical Analyses

Statistical analysis was performed with SPSS 17.0 software (SPSS, Inc., Chicago, IL). Data are presented as mean ± SD. Statistically significant differences were analyzed by one-way analysis of variance (ANOVA) and Newman–Keuls post hoc tests. A value of p < 0.05 was considered statistically significant, N=6.

## 3. Results

### 3.1. Gross Observation of Animals

No rabbits died, no implants failed and no peri-implantitis happened in this experiment. Food intake decreased and body weight dropped slightly after radiation, and they started to regain their weight within 2 weeks. In HBO group, no rabbits were found to suffer tympanic membrane rupture or other abnormalities. The shaved hair around the operation showed slower growth in irradiation group than in nonirradiation groups.

### 3.2. Implant Stability

**Figure 2:**
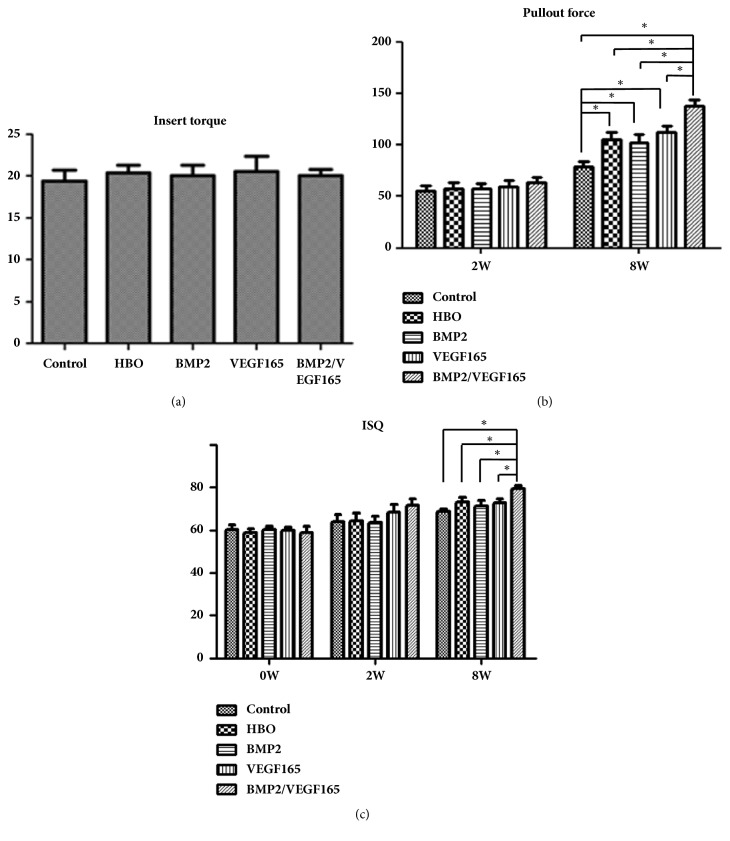
Stability of implants was measured by different methods at experimental time. (a) Insert torque. (b) Pullout force. (c) ISQ values. ^*∗*^P < 0.05. All values expressed as mean ± SD, N=5.

The insertion torque was shown in Figure 2(a). All of the implants had good stability and the torque was between 18NCM and 23NCM. There was no statistical discrepancy between the control group and the experimental groups. In the pullout test, no statistical difference was found between the control group and the experimental groups at two weeks (Figure 2(b)). However, at eight weeks, the pulling force in experimental groups was significantly higher than the force in the control group (P < 0.05). When compared to BMP2, VEGF-165, and HBO groups, the pulling force in BMP2/VEGF165 group was much higher (P < 0.05). There was no difference in implant stability quotient (ISQ) between the experimental groups and the control at two weeks (P > 0.05). At eight weeks, the four experimental groups showed higher ISQ than the control group. However, only BMP2/VEGF165 group showed statistical significance (P < 0.05) (Figure 2(c)).

### 3.3. Concentration of Serum Calcium and ALP Activity

**Figure 3:**
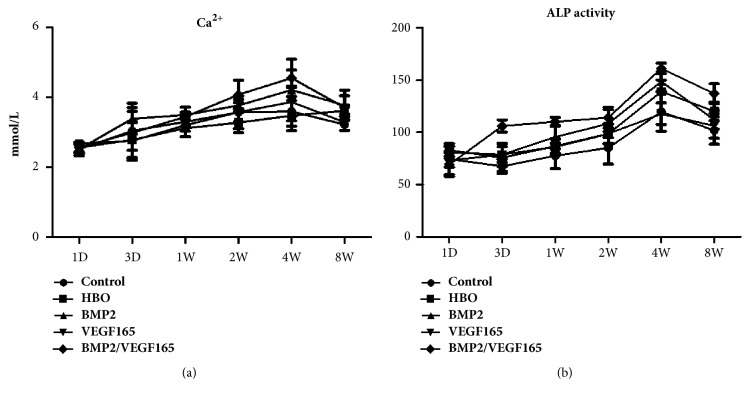
Ca^2+^ and ALP activity in serum were analyzed. (a) Concentration of Ca^2+^ in serum at different time. (b) ALP activity in serum.

The concentration of calcium and ALP activity in serum gradually increased in the first four weeks and dramatically declined at eight weeks after implant surgery in the five groups. In the first week, the concentration of calcium was higher in the four experimental groups than the control group, but no statistical difference was found (P > 0.05). At two weeks and four weeks, the concentration in BMP2 group and BMP2/VEGF165 group was significantly higher than in the control group (P < 0.05). At four weeks, the difference between the BMP2/VEGF165 group and HBO group showed statistical significance (p < 0.05). Alkaline phosphatase activity in serum in the five groups reached the peak at the fourth week. And in the BMP2 group and BMP2/VEGF165 group, it was significantly higher than in the other three groups (P < 0.05) (Figure 3).

### 3.4. Micro-CT Analyses

**Figure 4:**
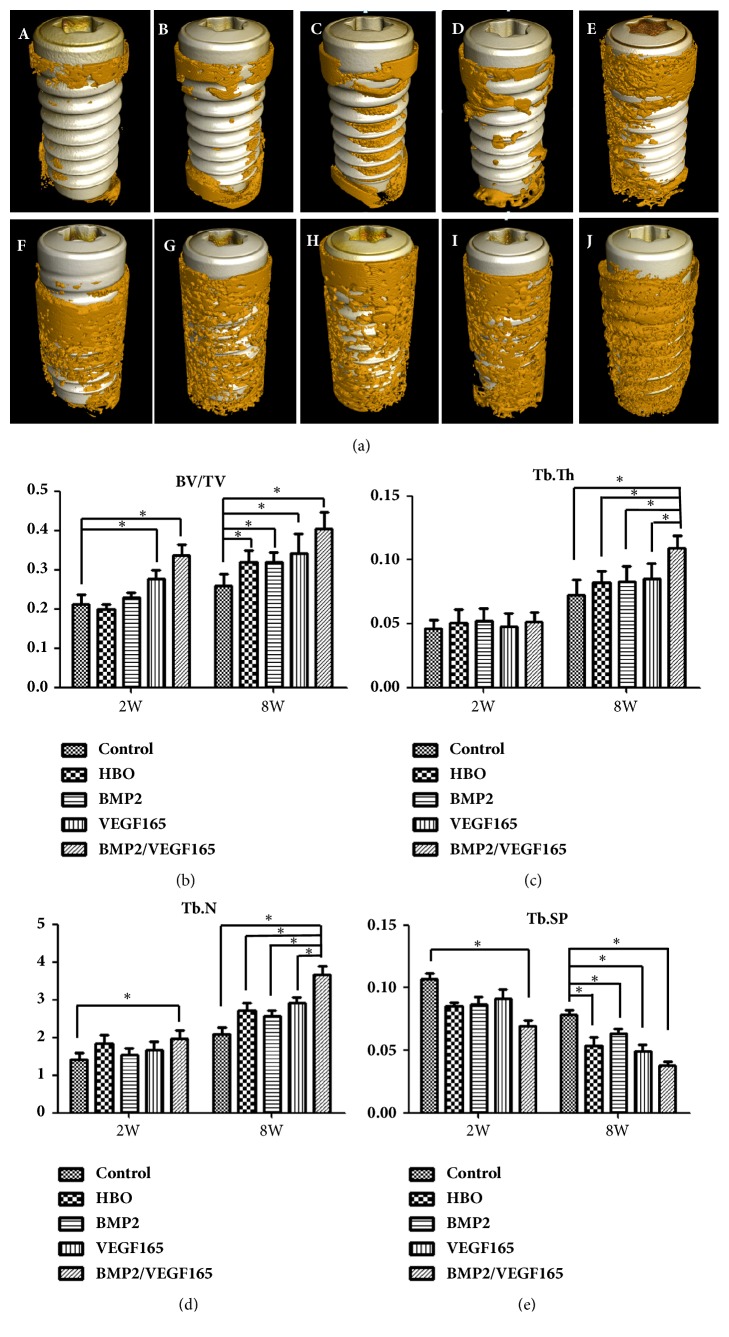
(a) 3D reconstructed images of implant and contacting bone tissues based on Micro-CT images. White color represents implants and yellow color represents bone tissues. (A) Control at 2 weeks; (B) HBO at 2 weeks; (C) BMP2 at 2 weeks; (D) VEGF165 at 2 weeks; (E) BMP2/VEGF165 at 2 weeks; (F) control at 8 weeks; (G) HBO at 8 weeks; (H) BMP2 at 8 weeks; (I) VEGF165 at 8 weeks; (J) BMP2/VEGF165 at 8 weeks; (b) BV/TV; (c) Tb.Th; (d) Tb.N; (e) Tb.Sp. ^*∗*^P < 0.05. All values are expressed as mean ± SD, N=5.

Three-dimensional reconstructed images of Micro-CT showed that bone tissue was visible on the surface of the implant, and there were also regions of low density that was not covered by bone tissue at two weeks and eight weeks after surgery (Figure 4). At two weeks, BV/TV in BMP-2/VEGF-165 group and VEGF-165 group was higher than that in the control group (P < 0.05). Then, Tb.Sp in the control group was wider than BMP2/VEGF165 group (P < 0.05). Tb.N in BMP2/VEGF165 group was higher than that in control group (P < 0.05). At eight weeks, BV/TV in the control group groups was lower than in the four experimental groups (P < 0.05). Tb.Th and Tb.N in group BMP2/VEGF165 were significantly higher than those in the control group, respectively. On the contrary, Tb.Sp in control group was much wider than in the four experimental groups (P < 0.05).

### 3.5. Fluorescence Observation

**Figure 5:**
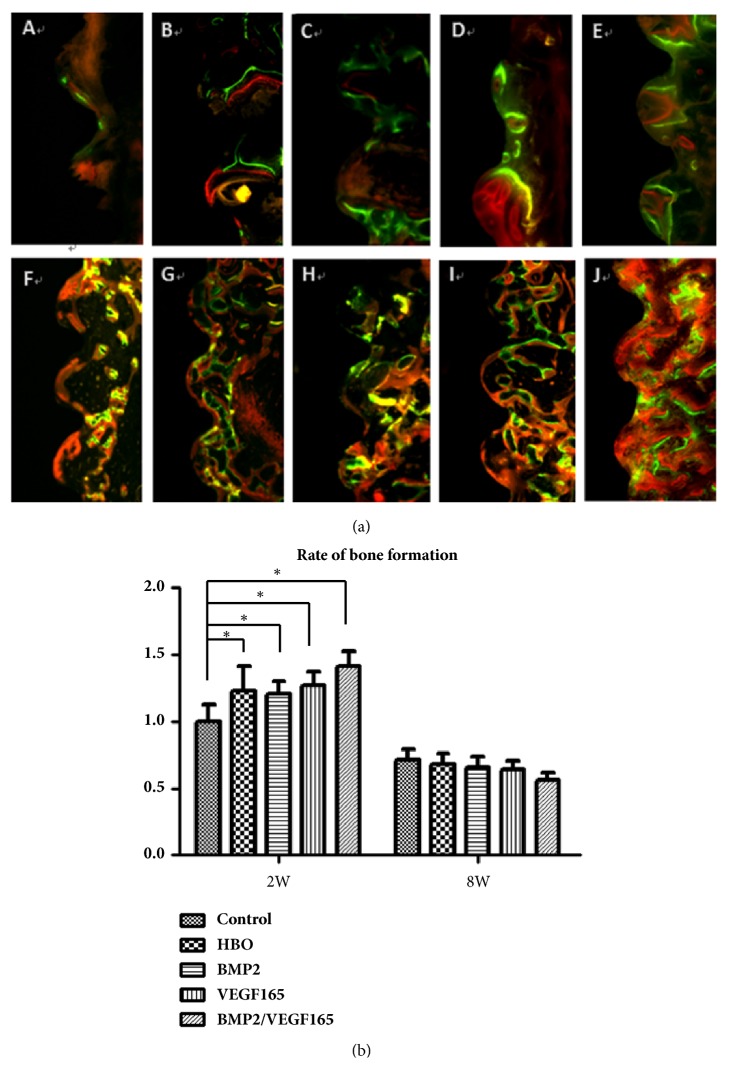
(a) Fluorochrome labeling images showing osseointegration under fluorescence microscopy. Red color was labeled by alizarin red at week 1, green color was labeled by calcein green at week 2, (A) control at 2 weeks; (B) HBP at 2 weeks; (C) BMP2 at 2 weeks; (D) VEGF165 at 2 weeks; (E) BMP2/VEGF165 at 2 weeks; (F) control at 8 weeks; (G) HBP at 8 weeks; (H) BMP2 at 8 weeks; (I) VEGF165 at 8 weeks; (J) BMP2/VEGF165 at 8 weeks; (b) rate of bone growth measured by fluorochrome labeling analysis. ^*∗*^P < 0.05. All values are expressed as mean ± SD, N=5.

The newly formed bone around the implant is marked by three different fluorescent colors: red is formed by alizarin red, green is formed by calcein, and yellow is formed by the combination of alizarin red and calcein (Figure 5). At two weeks, the deposition of fluorescent colors with clear stripes was seen in the four experimental groups. However, sporadic fluorescence was observed in the control group; they were in a disorderly arrangement and not clearly distinguishable. In BMP2/VEGF165 group, green bands and red bands of fluorescent strips were clearly visible, and the distance between them was larger than the other groups. At eight weeks, the fluorescent bands in the control group between threads of the implant were less and weaker than those in the experimental groups. More dense fluorescent bands were visible between the threads in BMP2/VEGF165 group than in other groups, and red fluorescence and green fluorescence were closely connected. The rate of bone growth was shown in Figure 5. At 2 weeks, the rate of bone deposition in the control group was significantly slower than that in the four experimental groups (P < 0.05). And the BMP-2/VEGF-165 group was faster than the other four groups (P < 0.05). At eight weeks, the rate of bone deposition in each group was significantly lower than that of two weeks (p < 0.05).

### 3.6. Real-Time RT-PCR Analyses

**Figure 6:**
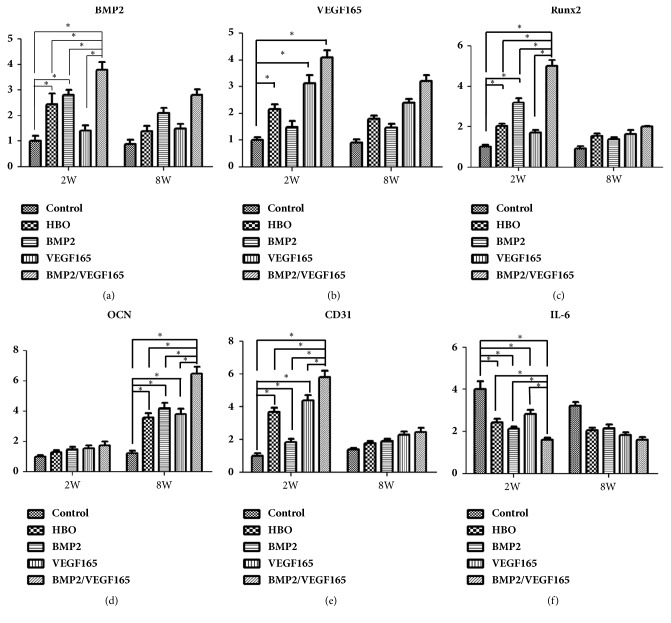
RT-PCR. (a) BMP2 expression; (b) VEGF165 expression; (c) RUNX2 expression; (d) OCN expression; (e) CD31 expression; (f) IL-6 expression. ^*∗*^P < 0.05. All values are expressed as mean ± SD, N=5.

The expression of angiogenesis-related gene, osteogenic related genes, BMP2, and VEGF165 was determined by qRT-PCR (Figure 6). In BMP-2 group, hyperbaric oxygen group, and BMP-2/VEGF-165 group, the expression of BMP2 was higher than that of the control group at 2 and 8 weeks (P < 0.05). At eight weeks, the expression of BMP2 in BMP2/VEGF165 group was the highest in the five groups (P < 0.05). The expression of VEGF165 in the control group was lower compared to the four experimental groups, but the difference was statistically significant only in HBO group, VEGF-165 group, and BMP-2/VEGF-165 group (P < 0.05). The expression of RUNX2 at 8 weeks was less than that of 2 weeks in the five groups. The expression of RUNX2 in the four experimental groups was significantly higher than in the control (P < 0.05), and it was highest in BMP2/VEGF165 group of the four experimental groups (P < 0.05). The expression of OCN at eight weeks was similar to that of RUNX2 at two weeks. At two weeks, compared to the control group, the expression of CD31 in the four experimental groups was higher (P < 0.05), and the BMP2/VEGF165 group was the highest in the experimental groups (P < 0.05). The expression of IL-6 was similar at the two and eight weeks. The control group was significantly upregulated relative to the other four groups (P < 0.05). And the BMP2/VEGF165 group was significantly lower than the other four groups (P < 0.05).

## 4. Discussion

Radiotherapy, one of the most effective treatments for cancer, could cause harm to the surrounding tissue, leading to many side effects, such as low cell activity, hypoxic concentration, and low blood vessel density [23]. These side effects would influence the process of osseointegration of titanium implants and thus increase the risk of implant surgery. Therefore, it is still a controversial topic whether it is suitable to place titanium implants in irradiated bone [8]. A recent study indicated that the impact that the restoration of normal oral function exerts on quality of life of patients with radiotherapy seems to outweigh the risks of the implant operation [24]. Although the success rate of titanium implants in nonirradiated bone was as high as 97%, it varied from 78% to 98% in irradiated bone in different studies [24]. Schoen's study showed that radiotherapy should not be considered an absolute contraindication for dental rehabilitation. It can easily lead to osteoradionecrosis in mandibular of patient receiving radiotherapy [25]. However, recent systematic reviews in humans concluded that the placement of implants in irradiated bone is viable, predictable, and reliable [24–26]. Similarly, our results also showed a high success rate both in experimental groups and control groups. However, the conclusion is based on the condition that the radiation dose is lower than 55 Gy and there is no force loading on titanium implants [8, 27].

Asikainen at al. found that the success rate of the implant decreased with the increase of radiation dose using a dog model, and 10% of the implants were lost and 40% of the implants showed the marginal bone resorption under 50 Gy radiation. When the radiation reaches 60 Gy, no implant survived and the supporting bone tissue was severely absorbed [11]. In our research, 15 Gy radiation was chosen as the experimental dose, as 15 Gy was equal to the overlay of the total dose of a clinical radiotherapy cycle and it also has been used in many studies in a rabbit model [11]. In accordance with other studies, the result showed that no implant was lost in our research under this radiation dose [8]. The key to the success of titanium implant is based on the osseointegration of bone and titanium implant, which could be seriously affected by radiation. Studies showed that the BIC was reduced significantly in irradiated bone [27, 28]. At 16 weeks after implant surgery, a 28% BIC was found in irradiated mandibles compared to 69% in nonirradiated mandibles [27, 28]. Li et al. found that radiation compromised osseointegration in a dose-dependent manner, which means radiation was adversely related to osseointegration [8]. To alleviate side effects of radiation, increase the osseointegration, and improve the success rate of titanium implant in irradiated bone, various methods were used. Hyperbaric oxygen (HBO), commonly used for the head and neck cancer patients, could increase local blood supply, local oxygen supply, and cytotoxicity. But HBO was not a statistically significant predictor for the implant survival and it did not have a statistically significant effect on the success rate of titanium implants in irradiated bone [26]. In our research, both radiation group and HBO group showed a high success rate of titanium implants and no one was lost. However, the BV/TV, Tb.N, and Tb.Th in HBO group were much higher than those in radiation group (Figure 5). And the bone growth rate showed the same trend. This means that HBO did not increase the success rate of the implant at the macrolevel, although it could promote the formation of new bone around the implant and osseointegration of implant at the microlevel. Radiotherapy could injury the remodeling system by damaging vascular endothelial cell and decreasing the proliferation of bone marrow and osteoblasts. Vascular injury is characterized by hyperemia and inflammation followed by endarteritis, vascular fibrosis, and microcirculation obstacle. The bone marrow injury showed signs of marked fibrosis and fatty degeneration for hypocellular and hypovascular [9]. HBO in irradiated tissue could increase tissue oxygen partial pressure intermittently to optimize the proliferation and differentiation of BMSC, osteoblast, and vascular endothelial cell, stimulate angiogenesis, and promote bone formation. HBO created a steep oxygen gradient across a short distance between normal and irradiated tissue, which triggers the recognition of radiated tissue as a wound and initiates angiogenesis and bone formation. Then the expression of osteogenic genes and vascular-related genes would increase accordingly [29]. Similar to our results, the expression of bone-related genes (RUNX2 and OCN) and vascular-related genes (CD31) significantly increased in HBO group compared to the irradiated group.

In addition, we analyzed the effect of VEGF165 and BMP2 in the osseointegration under radiation. Their effects were similar to HBO group. But the combined use of BMP2/VEGF165 showed a much better effect in osseointegration, compared with HBO group, BMP2 group, and VEGF165 group. As discussed above, radiation would cause vascular damage, induce local microcirculation disorder, and decrease the expression of VEGF165 [15, 16, 29]. Schliephake reported that VEGF165 immobilized on the surface of titanium implants by using a modular binding system could enhance the contact rate between the bone and the implant surface to a certain extent [30]. In our radiation model, the BV/TV, Tb.N, and Tb.Th around the titanium implant in VEGF165 group were much higher than those in radiation group, and the bone formation rate was faster in VEGF165 group. VEGF165 could promote angiogenesis, improve local microcirculation disorder, and alleviate the side effects of radiation [15, 16]. Kim and Sharmin found that an implant coated with BMP-2 could help bone regeneration and enhance the BIC area in the defect site around the implant without an additional bone graft, but some other studies showed that the combined delivery of VEGF165 and BMP2 for repair of critical size mandibular defects around the implant in vivo can significantly enhance quality and quantity of early and late bone formation over delivery of BMP2 or VEGF165 alone [18, 21]. On the one hand, in an early stage, VEGF165 could promote angiogenesis, increase local blood supply, and enhance the biological activity of local tissue. On the other hand, BMP2 could stimulate the differentiation of BMSC into osteoblasts, promote the secretion of collagen fibers from osteoblasts, accelerate bone deposition, help bone formation around the implant, and increase the BIC. During the formation of bone and the healing process of the titanium implant, the roles of VEGF165 and BMP2 are coordinated and mutually promoted [15, 17, 18, 21, 22]. In our irradiated model, the VEGF165/BMP2 group showed a better performance in the quality and quantity of bone formation, bone formation rate, and bone-implant contact around the implant compared to HBO, VEGF165, or BMP2 alone.

ISQ value, a key factor used for determining different healing phases, the stability of the implants, bone density, the area of bone-implant contact, and the appropriate time of implant loading, could be assessed during osseointegration [31–35]. It decreased in the first month after implant was inserted and then increased in the second and third month, suggesting an adaptive bone remodeling process around the implant and being used for detecting minor changes of BIC [32, 36]. The result showed that ISQ values were lower in two weeks than in eight weeks in all groups. Since radiotherapy could significantly reduce bone vascularity, bone density, bone volume, bone regenerative capability, and implant osseointegration, the reduction in ISQ values was more pronounced in irradiated compared to that in nonirradiated alveolar bone [32–34]. In our experiment, the ISQ values and pullout values of the VEGF165 group, BMP2 group, HBO group, and BMP2/VEGF165 group were remarkably higher than those of the control group, and the BMP2/VEGF165 group achieved maximum value. It means that HBO, BMP2, and VEGF165 could help to promote bone formation around the implant, improve quality and quantity of bone, increase BIC, and lower the effect of radiation on the implant in irradiated bone. And the effect of combined use of BMP2 and VEGF165 would be better than HBO or single use.

In conclusion, HBO, BMP2, and VEGF165 could increase bone formation around the implant, promote BIC, and develop the implant stability in irradiated bone. And the combined use of BMP2 and VEGF165 achieved the best effect on these aspects. The result of this phenomenon provides a beneficial reference for improving the success rate of titanium implants and the quality of life for patients with radiotherapy. The clinical significance needs to be elucidated in future studies.

## Figures and Tables

**Table 1:** The primer pairs were used in the study.

Gene	Primer forward	Primer reverse
GAPDH	5'-AGAACAGAGTCATCCCACAC-3'	5'--GCTACGTTATTCTTGCCATC-3'
BMP2	5' TGGAATGACTGGATTGTGGCT-3'	5'- CTATCGTGACTCAAGACAGCCCT-3'
VEGF165	5' GATGAGCTTCCTACAGCACAACAA-3'	5'- GTTTACGAAAGAGGCGAGACT-3'
OCN	5'- GTGCTGAATCCCGCAAAGG -3'	5'- CATACTTCCCTCTTGGGCTCC-3'
RUNX2	5'- GACCAGCAGCACTCCATATC -3'	5'- CATCAGCGTCAACACCATCATTC-3'
CD31	5'-AACAGTGTTGACATGAAGAGCC-3'	5'-TGTAAAACAGCACGTCATCCTT-3'
IL6	5'-TGGCTGAAGACGACCACGAT -3'	5'-TTCCGCAAGCAAGGACACC -3'

## Data Availability

The data used to support the findings of this study are available from the corresponding author upon request.
